# Supplementary material for: MetaRibo-Seq measures translation in microbiomes
Source: Nat Commun. 2020 Jun 29;11:3268. doi: 10.1038/s41467-020-17081-z (PMC7324362; doi:10.1038/s41467-020-17081-z)
Supplement: Supplementary file 10 — Supplementary Data 7 [file 41467_2020_17081_MOESM10_ESM.zip › File2/Confidence_High_Taxonomy/108625_out.krona.html]

Javascript must be enabled to view this page.

members
magnitude
magnitudeUnassigned
count
unassigned
taxon
rank

108625\_out

11

11
2
superkingdom

phylum
11
976

200643
11
class

order
11
171549

family
11
815

genus
11
816

species

SRS019496\_contig\_number\_6254SRS048164\_contig\_number\_11955SRS104693\_contig\_number\_715
821
3

species

SRS016381\_contig\_number\_2503SRS024435\_contig\_number\_25439
2292923
2

357276
6

SRS012273\_contig\_number\_29940SRS016018\_contig\_number\_10509SRS042628\_contig\_number\_32991SRS048981\_contig\_number\_843SRS075021\_contig\_number\_426SRS1041112\_contig\_number\_5118
species
